# High-Dose Levomethadone in Opioid Dependence: Case Study Exploring Pharmacogenetics and Management of Benzodiazepine Withdrawal

**DOI:** 10.1192/j.eurpsy.2025.991

**Published:** 2025-08-26

**Authors:** M. F. Melloni, P. Boldrini Parravicini Persia, F. Cappella, F. Odorizzi, R. P. Sant’Angelo

**Affiliations:** 1 Psychiatry, University of Bologna, Bologna; 2 Mental Health Department, Ausl Romagna, Cesena, Italy

## Abstract

**Introduction:**

Methadone is typically administered as a racemic mixture of two enantiomers (50% Dextro, 50% Levo methadone) and is used for chronic pain management and as maintenance therapy for opioid dependence. Levomethadone, when used alone, shows similar efficacy but with fewer side effects, particularly a safer cardiac profile with less QTc prolongation. In maintenance therapy for heroin dependence, the effective dosage of levomethadone ranges from 40 to 70 mg per day.

**Objectives:**

Our case concerns a 42-year-old patient who has struggled with substance abuse since the age of 17. Admitted to the Psychiatric Unit of Cesena Hospital for a reduction of Lormetazepam (previously consuming 80-100 mg/day), he was found to be taking 200 mg of levomethadone daily. This dosage is typically administered for pain management rather than as maintenance therapy for heroin dependence. Although the man was consistently consuming high doses of psychoactive medications, exceeding the ranges commonly reported in the literature, he did not exhibit significant adverse effects or signs of sedation during his hospital stay. This observation led us to consider the possibility of a genetic alteration in cytochrome enzymes that could enable ultra-rapid drug metabolism.

**Methods:**

During the hospital stay, it was possible to safely reduce the benzodiazepine therapy by switching from oral lormetazepam to intravenous diazepam and subsequently to oral diazepam.The patient also underwent a pharmacogenetic test that analyzes the polymorphisms of 60 different enzymes using cells obtained from saliva.

**Results:**

In the reported case, the use of L-Methadone has allowed over the years a full control of withdrawal symptoms and cravings from opioid drugs, a greater compliance with treatment and a lower risk of general and cardiological side effects than racemic methadone administered in equivalent therapeutic doses in past years.

It has also been made possible, through in-patient treatment, to carry out a progressive withdrawal from Lormetazepam in total safety.

Pharmacogenetic testing targeting CYP3A4 and CYP2B6 enzymes did not reveal significant alterations, contradicting our initial hypothesis.

**Conclusions:**

The originality of this case is basically due to the lack in the literature about clinical cases treated with such a high dose of Levomethadone (200 mg/day) as substitution therapy for opioid addiction and to the investigation of the salivary pharmacogenetic testing to eventually support the hypothesis that the patient could be a rapid or ultrarapid metabolizer.

Moreover, we have sought to clarify the correct use of levomethadone in individuals at high risk of death due to conditions that may increase the risk of Torsades de Pointes.

The pharmacogenetic analysis excluded rapid metabolism, suggesting a role for P-glycoprotein (PGP) in influencing the absorption of methadone and the variability of plasma concentration.

**Disclosure of Interest:**

None Declared

